# Supplementary figures and images for: Pangenomic Characterization of Campylobacter Plasmids for Enhanced Molecular Typing, Risk Assessment and Source Attribution
Source: Pathogens. 2025 Sep 16;14(9):936. doi: 10.3390/pathogens14090936 (PMC12472675; doi:10.3390/pathogens14090936)

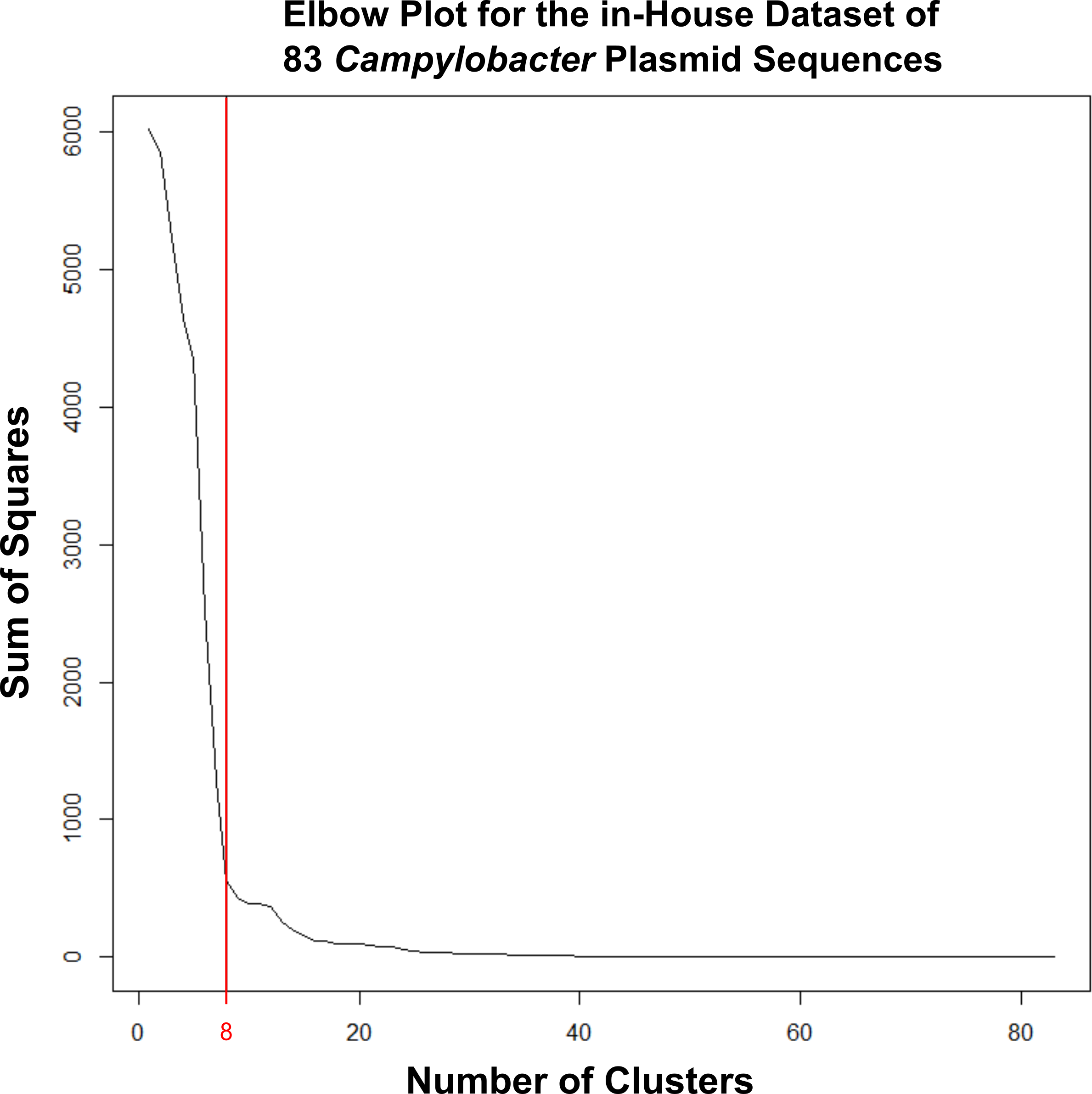

Supplement: Supplementary file 1 [file pathogens-14-00936-s001.zip › Figure_S1.png]

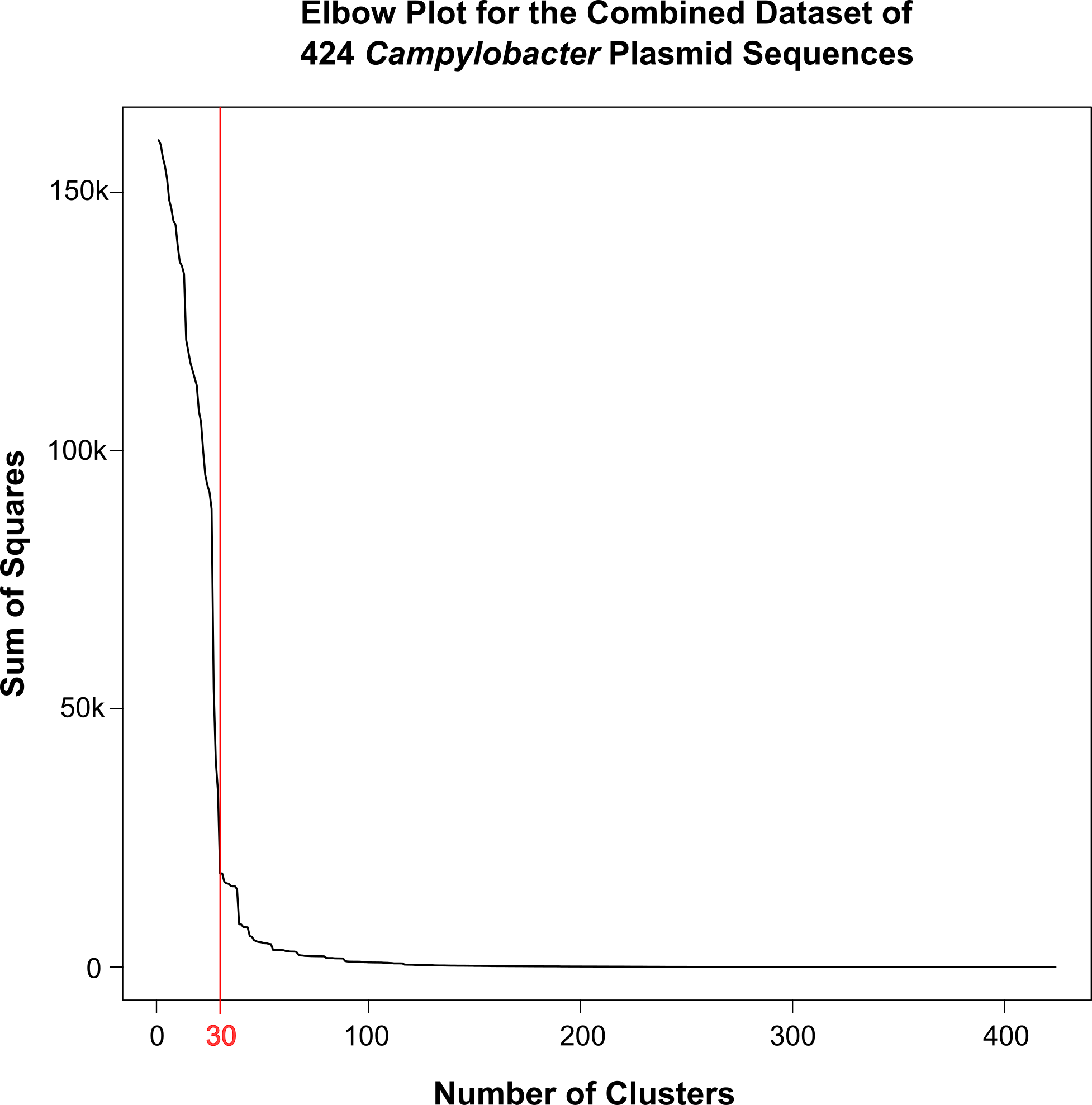

Supplement: Supplementary file 1 [file pathogens-14-00936-s001.zip › Figure_S2.png]

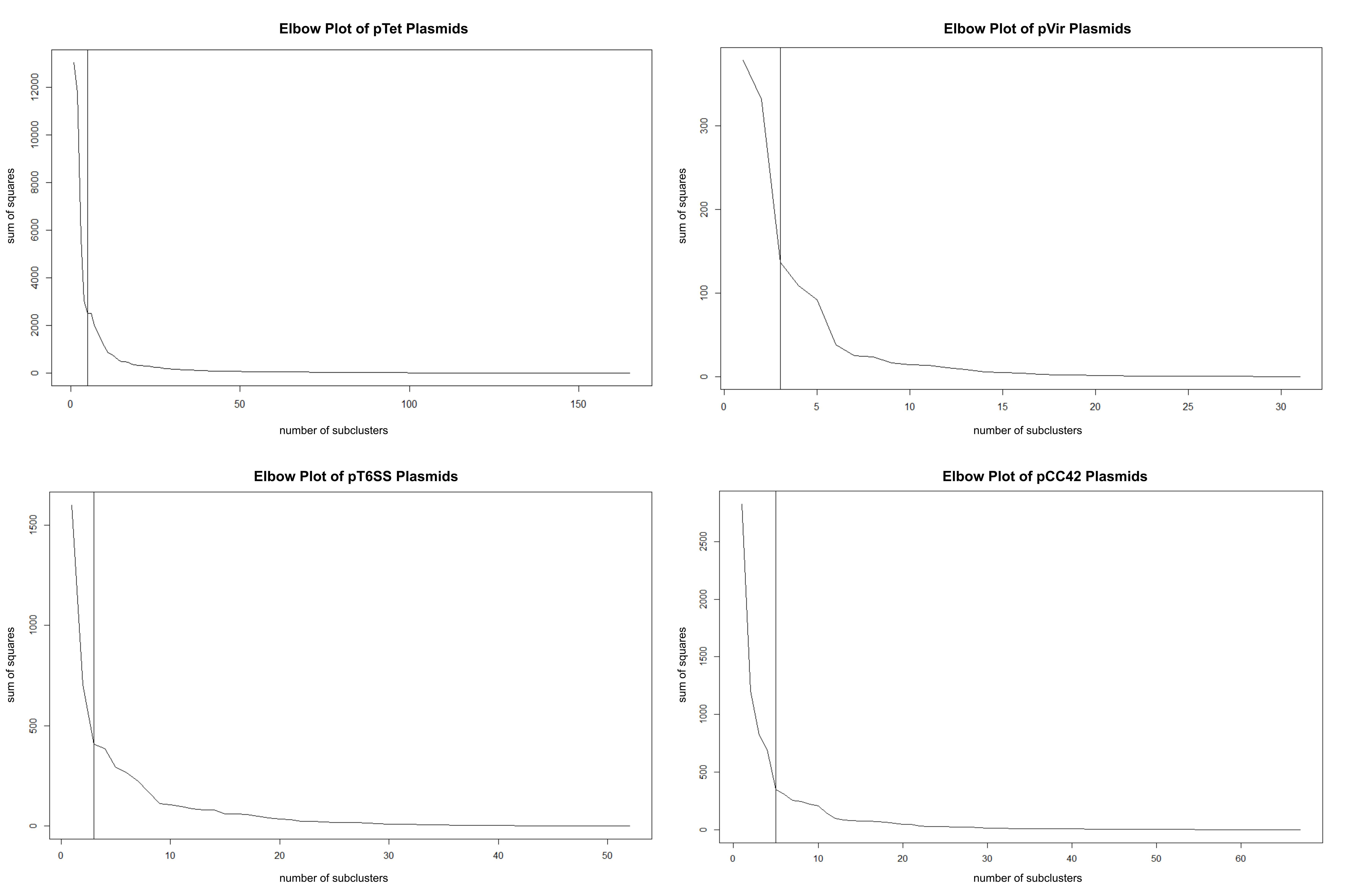

Supplement: Supplementary file 1 [file pathogens-14-00936-s001.zip › Figure_S3.png]

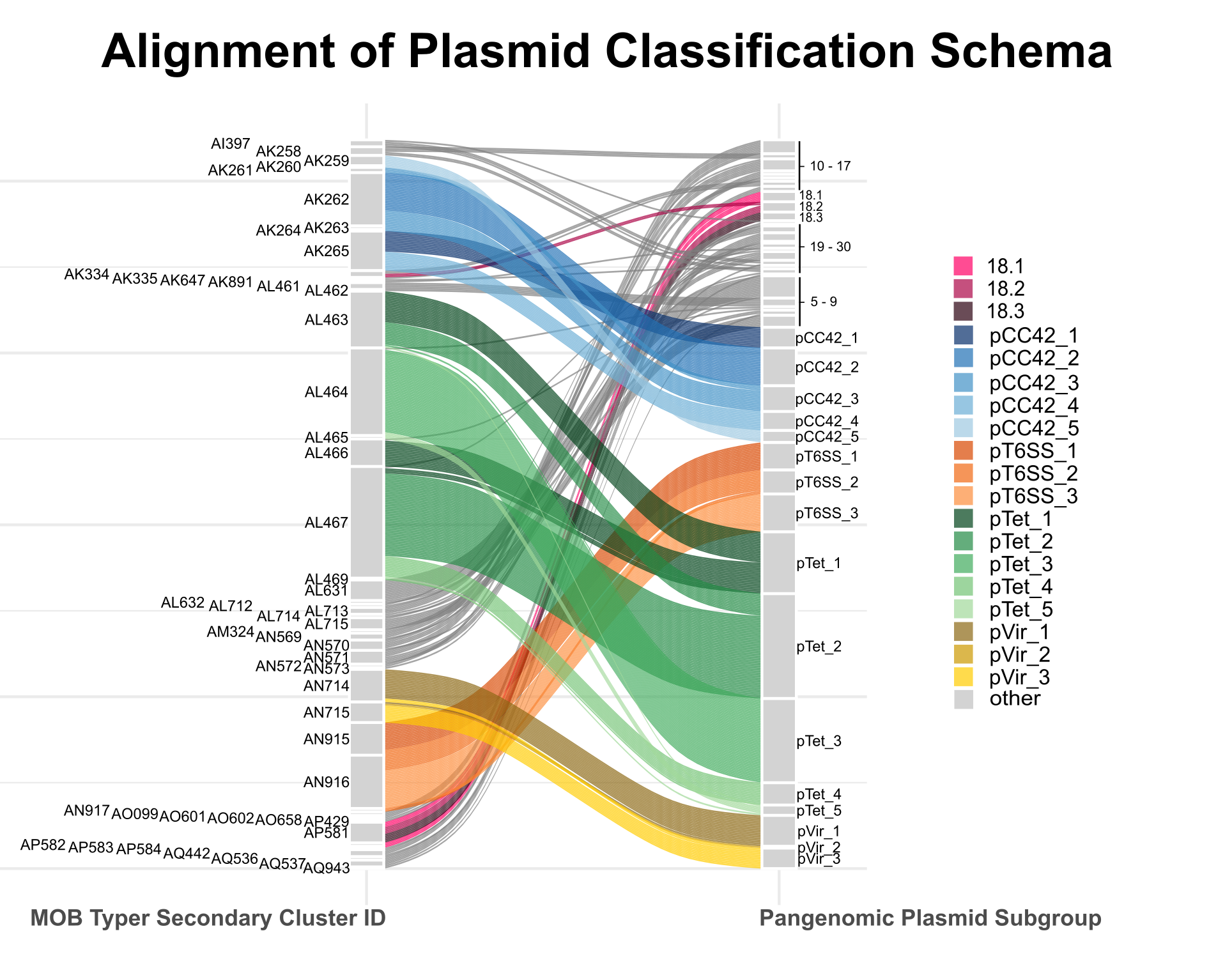

Supplement: Supplementary file 1 [file pathogens-14-00936-s001.zip › Figure_S4.png]

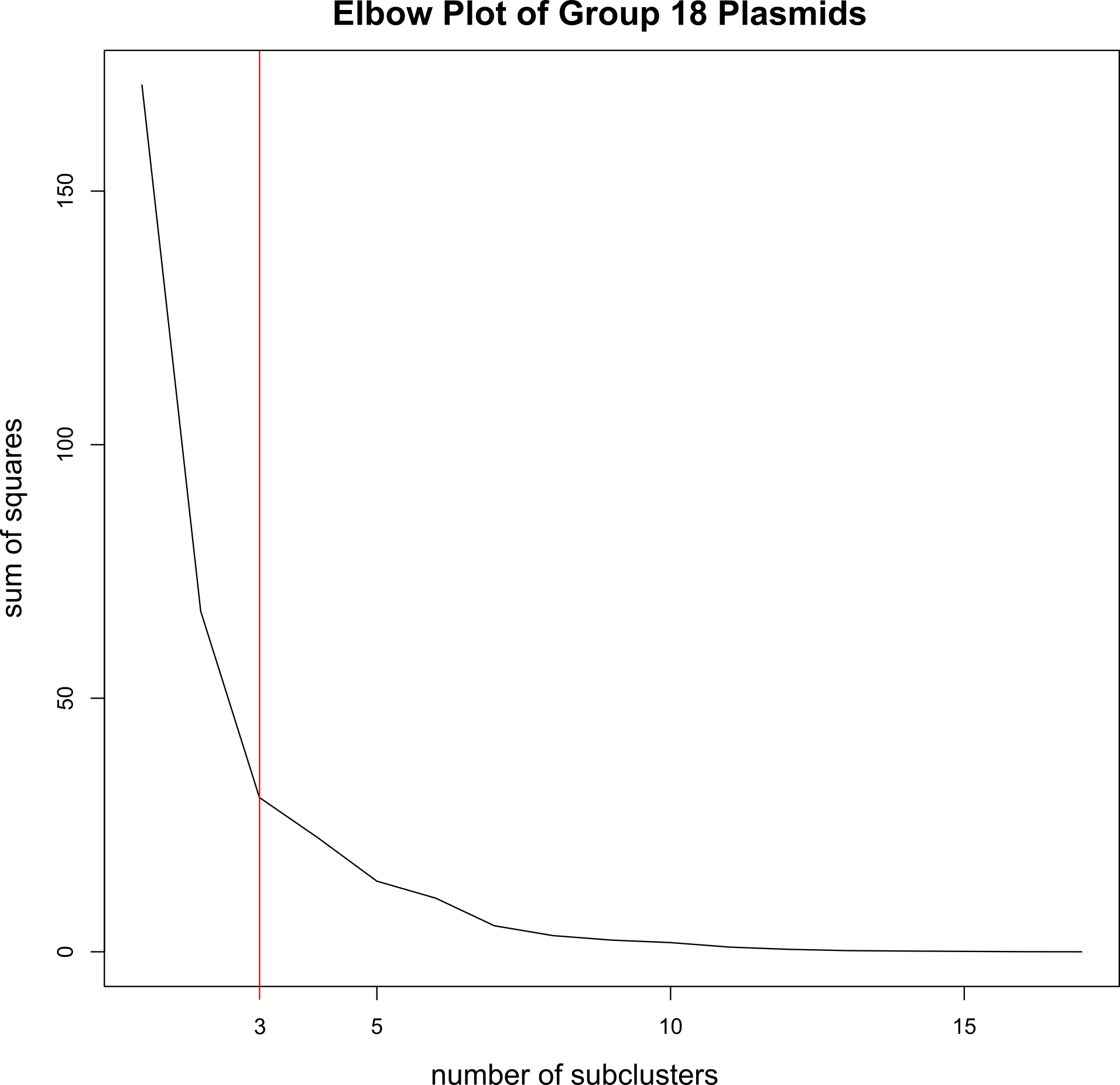

Supplement: Supplementary file 1 [file pathogens-14-00936-s001.zip › Figure_S5.png]
